# Extracorporeal carbon dioxide removal for acute hypercapnic respiratory failure

**DOI:** 10.1186/s13613-019-0551-6

**Published:** 2019-07-02

**Authors:** Luis Morales-Quinteros, Lorenzo Del Sorbo, Antonio Artigas

**Affiliations:** 1 Intensive Care Unit, Hospital Universitario Sagrado Corazón, Barcelona, Spain; 2 Interdepartmental Division of Critical Care Medicine, Toronto General Hospital, University of Toronto, Toronto, Canada; 3 Critical Care Center, ParcTaulí Hospital Universitari, Institut d’Investigació i Innovació Parc Taulí I3PT, Universitat Autònoma de Barcelona, Sabadell, Spain; 4 CIBER Enfermedades Respiratorias, Instituto de Salud Carlos III, Madrid, Spain

**Keywords:** COPD, Asthma, ECCO_2_R, Invasive mechanical ventilation, Noninvasive mechanical ventilation

## Abstract

In the past, the only treatment of acute exacerbations of obstructive diseases with hypercapnic respiratory failure refractory to medical treatment was invasive mechanical ventilation (IMV). Considerable technical improvements transformed extracorporeal techniques for carbon dioxide removal in an attractive option to avoid worsening respiratory failure and respiratory acidosis, and to potentially prevent or shorten the duration of IMV in patients with exacerbation of COPD and asthma. In this review, we will present a summary of the pathophysiological rationale and evidence of ECCO_2_R in patients with severe exacerbations of these pathologies.

## Background

Patients with obstructive lung diseases, such as asthma and chronic obstructive pulmonary disease (COPD), may experience acute exacerbations with severe hypercapnic respiratory failure. Hypercapnia results from acute worsening of expiratory flow limitation caused by the increased small airway resistance with consequent development of dynamic alveolar hyperinflation and intrinsic positive end-expiratory pressure (PEEP). In the most severe cases, these may be refractory to conventional therapies and mechanical ventilation, becoming life-threatening.

Extracorporeal carbon dioxide removal (ECCO_2_R) represents an attractive approach in this setting.

The last decade has seen an increasing interest in the provision of extracorporeal support for respiratory failure, as demonstrated by the progressively increasing number of scientific publications on this topic. In particular, remarkable interest has been focused on extracorporeal carbon dioxide removal (ECCO_2_R), due to the relative ease and efficiency in blood CO_2_ clearance granted by extracorporeal gas exchangers as compared to oxygen delivery [1].

In recent years, a new generation of ECCO_2_R devices has been developed. More efficient veno-venous (VV)-ECCO_2_R devices have become available and have replaced the arterio-venous approach, having the advantage of not requiring arterial puncture.

The new VV-ECCO_2_R devices offer lower resistance to blood flow, have smaller priming volumes, and provide a much more efficient gas exchange with relatively low extracorporeal blood flows (0.4–1 L/min) [2]. The technology of these devices is now comparable to that of renal dialysis and has been experimented in several animal and human studies, demonstrating significant reduction in arterial CO_2_ and improvement in the work of breathing [3–6].

## Pathophysiological rationale for ECCO_2_R in obstructive lung diseases

In both asthma and COPD exacerbations, diffuse narrowing of the airways results in detrimental physiological consequences. Airway narrowing prevents the lungs from completely emptying (“air trapping”) due to resistance to expiratory flow and bronchial closure at higher than normal lung volumes. Air trapping results in dynamic hyperinflation (DHI) [7] which is the excessive increase in end-expiratory lung volume above the relaxation volume of the respiratory system, generating intrinsic positive end-expiratory pressure (auto-PEEP) [8]. As a result, the patient breathes at higher total lung volumes, due to increased residual volume [9], which may reduce tidal ventilation. The net effect is that the work of breathing increases significantly. The diaphragm, intercostal muscles, and even the abdominal muscles are overloaded causing respiratory muscle fatigue and dyspnea [10].

Pharmacotherapy with bronchodilators and systemic corticosteroids are part of the medical therapies, administered specifically to reduce the pathophysiological airflow obstruction and improve symptoms.

The recognition for the need for noninvasive ventilation (NIV) is indicated if the patient fails to improve clinically and if the level of pH remains less than 7.32 despite medical therapy [11]. However, NIV fails in up to 20–30% of patients and IMV is indicated with specific ventilation strategies, targeting relative short inspiratory time and longer expiratory time [12, 13].

Overall, the goal of mechanical ventilation is to provide adequate gas exchange and reduce the work of breathing while waiting for airflow obstruction to resolve. However, mechanical ventilation itself may aggravate alveolar hyperinflation by worsening DHI, which may lead to worsened hypercapnia, barotrauma, alveolar rupture leading to pneumothorax and further hemodynamic deterioration [14].

Furthermore, if treated with IMV, these patients receive sedatives and likely neuromuscular blockade to facilitate ventilatory support [15]. Sedation and paralysis preclude mobilization, promoting neuromuscular deconditioning, and potentially contributing to the long-term cognitive sequelae of critical illness [16].

When conventional therapeutic options are not successful, novel therapies such as extracorporeal life support are entertained as a possible salvage therapeutic modality.

During exacerbation, relieving the native lung from at least part of the CO_2_ elimination with ECCO_2_R could potentially improve the acid–base balance, reduce patient’s work of breathing with a consequent reduction in respiratory rate and ventilatory drive, and lower alveolar ventilation. The lower tidal volumes and respiratory rate result in the extension of the expiratory time, suiting better the high expiratory time constant of the respiratory system with expiratory flow limitation. By these physiological mechanisms, ECCO_2_R can counteract the vicious circle of dynamic hyperinflation, and its detrimental respiratory and cardiovascular consequences. The derived beneficial effects on respiratory mechanics, ventilatory muscle efficiency, work of breathing, and cardiovascular function may improve gas exchanges and relieve dyspnea. By these mechanisms, ECCO_2_R thus can potentially prevent NIV failure, facilitate weaning from IMV, and therefore contribute to avoid the unwanted complications of sedation and immobilization.

## ECCO_2_R technical aspects and principle

ECCO_2_R is designed to remove carbon dioxide (CO_2_) and, unlike extracorporeal membrane oxygen (ECMO), does not provide significant oxygenation.

**Fig. 1:**
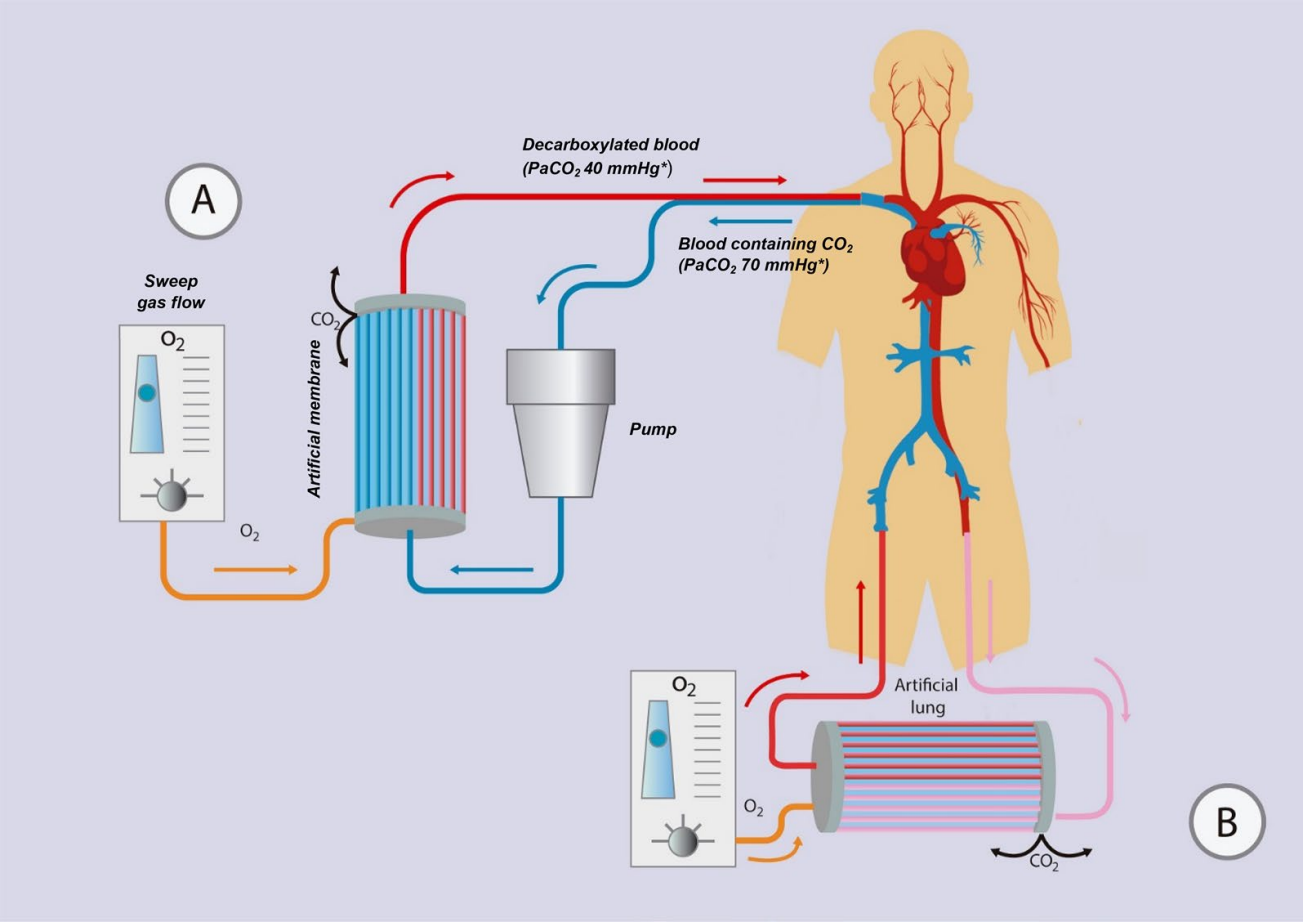
ECCO_2_R common configurations. **a** Minimally invasive veno-venous ECCO_2_R system with a single venous vascular access through a double-lumen cannula that can be inserted in the internal jugular or femoral vein. **b** Pumpless arterio-venous ECCO_2_R system with the placement of the membrane in the circuit connecting the femoral artery with the contralateral vein. *PaCO_2_ values are purely indicative

The device consists of a drainage cannula placed in a large central vein or artery, a membrane lung (artificial gas exchanger), and a return cannula into the venous system (Fig. 1). Blood is pumped through the membrane lung, and CO_2_ is removed by diffusion. A flowing gas known as “sweep gas” containing little or no CO_2_ runs along the other side of the membrane, ensuring a diffusion gradient from blood to the other side, hence promoting CO_2_ removal.

In contrast to ECMO, where the need for oxygenation requires high blood flow rates, ECCO_2_R requires much lower blood flow rates, due to the significant differences in CO_2_ and oxygen (O_2_) kinetics. Almost all the O_2_ in blood is carried by hemoglobin, which displays sigmoidal saturation kinetics. Assuming normal hemoglobin and venous O_2_ content, each liter of venous blood can only carry an extra 40–60 mL of O_2_ before the hemoglobin is fully saturated. Blood flows of 5–7 L/min through the extracorporeal artificial membrane lung are therefore required to supply enough O_2_ for an average adult. Conversely, most CO_2_ in blood is dissolved or in the form of bicarbonate, displaying linear kinetics without saturation. Considering that 1 L of blood is transported around 500 mL of CO_2_, in a perfectly efficient system flow of 0.5 L/min would be enough to remove all of the CO_2_ produced by an average adult, which is about 250 mL/min [2, 17, 18]. Also, CO_2_ diffuses more readily than O_2_ across extracorporeal membranes because of higher solubility. However, in practice, ECCO_2_R is usually able to remove up to 25% of carbon dioxide production given the limitations of blood flow and membrane efficiency [19]. As the rate of CO_2_ clearance greatly depends on the fresh sweep gas flow through the membrane lung, this is usually maximized in the low blood flow ECCO_2_R systems. Therefore, the efficiency of CO_2_ clearance of the different available devices is critically determined by other important parameters, including the size of the cannula, the rate of recirculation of blood in the circuit, the efficiency of the pump, the total surface area of the artificial lung, and the cardiac preload [20, 21]. Importantly, given the low blood flow through the extracorporeal circuit and the even lower flow achieved when the blood is crossing the large surface area of the artificial gas exchanger, the risk of thrombosis plays also a key role in CO_2_ clearance.

### VV-ECCO_2_R

In the veno-venous configuration, blood is drawn from a central vein by a draining cannula, using a centrifugal or roller pump to generate flow across the membrane. CO_2_ is removed by the effect of the “sweep gas,” and blood is then returned into the venous circulation (Fig. 1a). Single site cannulation is possible using a double-lumen cannula. This approach allows ECCO_2_R through the use of smaller cannulas (15-19F), commonly introduced via the right internal jugular vein. The setup is very similar to renal replacement therapy, and in fact, some systems are trying to combine the two in one [22, 23] (NCT02590575). One of the advantages of VV-ECCO_2_R compared to the AV approach is the less invasiveness by avoiding arterial cannulation, and the potential for early mobilization of patients. It is also possible to set up an ECCO_2_R system through cannulation of two central veins, one for drainage and the other for reinfusion (e.g., femoral–femoral configuration).

### AV-ECCO_2_R

In the AV-ECCO_2_R configuration, the blood flows from the femoral artery, usually instrumented with percutaneous cannulation, to the contralateral femoral vein, creating a pumpless arterio-venous (AV) bypass, equipped with an artificial gas exchanger across the AV shunt, which allows the “sweep gas” to remove CO_2_ (Fig. 1b). This pumpless systems require an arterio-venous pressure gradient ≥ 60 mmHg and a cardiac index > 3 L/min/m^2^, which is unsuitable for hemodynamically unstable patients [18, 24]. Furthermore, cannulation of a major artery can result in distal ischemia [25], although measuring the artery diameter with ultrasound and selecting a cannula that occupies less than 70% of the lumen reduces this risk [26].

## Indications and evidence

### Chronic obstructive pulmonary disease

Chronic obstructive pulmonary disease (COPD) is a significant worldwide health burden. Currently, it is the fourth leading cause of death worldwide, and is the only leading cause of death that is rising, and will likely become the third cause of death by 2020 [27, 28].

Acute exacerbations of COPD (aeCOPD) constitute a significant cause of morbidity and mortality among these patients. Patients with moderate to severe acute exacerbations develop alveolar hyperinflation that may lead to increased work of breathing, muscle fatigue, and hypercapnia, creating a vicious loop refractory to medical treatment [29–31]. The standard respiratory support in this setting is NIV, which however, fails in up to 30% of patients with aeCOPD, prompting intubation and IMV [32–34]. In recent meta-analysis and observational studies, it has been reported that the in-hospital mortality of patients with aeCOPD requiring IMV is as high as 25–39% [35–38].

Patients with COPD requiring IMV develop a considerable reduction in respiratory muscle strength, with higher risk of prolonged weaning and/or failure to wean, compared to other causes of acute hypercapnic respiratory failure. Up to 60% of the ventilatory time in these patients is spent for weaning [39] and is very likely to require a tracheotomy. The prolonged time on IMV results in an increased potential risk of ventilator-induced lung injury, ventilator-associated pneumonia, and ventilator-induced diaphragmatic dysfunction, in addition to the above-mentioned complications associated with prolonged sedation and immobilization.

#### Evidence and clinical trials of ECCO_2_R in aeCOPD to date

One of the first reports on the application of ECCO_2_R to support respiratory function of a COPD patient was published in Pesenti et al. [40]. However, the technique was abandoned due to technical complications.

As the medical community regained interest in ECCO_2_R, investigators began applying the technique to prevent intubation or to assist weaning from IMV in this patients’ population. Several studies in both VV and AV configurations were published, including a meta-analysis (Table 1).

**Table 1:** Relevant clinical studies of ECCO_2_R in COPD

References	No. of patients	ECCO_2_R characteristics				Time on ECCO_2_R	Major results
		Configuration	Blood flow (mL/min)	Sweep flow (L/min)	Membrane (material); surface in m^2^		
ECCO_2_R to avoid mechanical ventilation							
Kluge et al. [5]	21	Femoral AV with 13- to 15-Fr arterial cannula and 13- to 17-Fr venous cannula	1100	Not reported	PMP; 1.3 (iLA^®^)	9 days	19 (90%) PECLA patients did not require intubation Two major and seven minor bleeding complications during PECLA No significant difference in 28-day (24 vs. 19%, *p *= 0.85), 6-month mortality (33 vs. 33%), or hospital length of stay (23 vs. 42 days, *p *= 0.06) Significantly fewer tracheostomies in PECLA group (10 vs. 67%, *p *= 0.004)
Del Sorbo et al. [4]	25	Modified continuous VV hemofiltration system with membrane lung via 14-Fr single dual-lumen cannula (femoral)	255	8	PLP; 1.35 (Hemodec DecapSmart^®^)	1–2 days	Significantly higher risk of intubation in NIV-only group (HR 0.27; 95% CI 0.07–0.98) 13 patients experienced adverse events: three had bleeding, one had vein perforation, and nine had device malfunction
Braune et al. [43]	25	VV configuration via a 22 or 24-Fr single dual-lumen cannula (femoral or jugular)	1300	Not reported	PMP; 1.3 (Novalung iLA Activve)	8.5 days	Intubation was avoided in 14 out of all 25 ECCO_2_R patients (56%) Seven ECCO_2_R patients were intubated because of progressive hypoxemia and four due to ventilatory failure despite ECCO_2_R and NIV Nine ECCO_2_R patients (36%) suffered from major bleeding complications 90-day mortality rates were 28 vs. 28%

Study	No. of patients	ECCO_2_R characteristics				Time on ECCO_2_R	Major results
		Configuration	Blood flow (mL/min)	Sweep flow (mL/min)	Membrane surface (m^2^)		
ECCO_2_R to facilitate liberation from mechanical ventilation							
Abrams et al. [3]	5	VV configuration via a 20- to 24-Fr single dual-lumen jugular catheter using lower flow on ECMO system	1700	1–7	PMP; 0.98 (Maquet PALP CardioHelp)	8 days	Mean (SD) time to ambulation after ECCO_2_R initiation was 29.4 ± 12.6 h Four patients were discharged home, and one underwent planned lung transplantation Only two minor bleeding complications
Cardenas et al. [46]	1	VV configuration with pediatric dual-lumen jugular cannula	800	10	PMP; 1.8 (Quadrox-d, Maquet)	3.6 days	Patient extubated 48 h after decannulation. No complications reported
Roncon et al. [47]							
ECCO_2_R with mixed indications							
Burki [42]	20	VV configuration via a 15.5-Fr single dual-lumen catheter (femoral or jugular)	430	Not reported	PLP with a base of siloxane layer; 0.59 (ALung Hemolung RAS)	2–192 h	20 hypercapnic COPD patients received ECCO_2_R in three distinct groups: group 1 (*n *= 7) NIV patients with high risk of IMV; group 2 (*n *= 2) could not be weaned from NIV; and group 3 (*n *= 11) on IMV and failed to wean IMV avoided in all patient in group 1 Both patients in group 2 weaned from NIV In group 3, three patients weaned, and IMV was reduced in two patients One patient died due to a retroperitoneal hemorrhage (during cannulation)

*PMP* poly-4-methyl-1-pentene, *PLP* polypropylene

### ECCO_2_R to avoid IMV

Brederlau et al. [41] described their experience in three patients that failed NIV for severe aeCOPD. They applied a pumpless AV ECCO_2_R device with the goal of avoiding endotracheal intubation. The ECCO_2_R flow ranged between 1.1 and 1.6 L/min, with the sweep gas flow varying from 3 to 10 L/min. Shortly after beginning ECCO_2_R, the PaCO_2_ fell significantly, and also the respiratory rate dropped from 38, 45, and 37 breaths/min to 15, 25, and 18 breaths/min, respectively.

Kluge et al. [5] in the same year evaluated the safety and efficacy of using an AV pumpless extracorporeal lung assist in 21 COPD patients who did not respond to NIV and compared them to 21 matched controls treated with IMV. The use of AV ECCO_2_R resulted in the decrease of PaCO_2_ after 24 h and obviated the need for IMV in 90% of the experimental arm. Although the experimental group had a shorter hospital length of stay, there was no significant difference in mortality at 28 days (19% with ECCO_2_R vs. 24% without ECCO_2_R) or 6 months (both groups 33%) compared to the control group.

Burki et al. [42] treated 20 hypercapnic COPD patients with VV ECCO_2_R through a 15.5-Fr dual-lumen cannula achieving a mean blood flow of 430 mL/min. Of the 20 patients, seven were at risk of failing NIV, two were difficult to wean from NIV, and 11 had failed liberation from MV. With ECCO_2_R, none of the patients failing NIV required endotracheal intubation, and both patients with difficult weaning from NIV were weaned. However, only three of the 11 IMV patients were liberated successfully. Moreover, significant complications arose in a number of patients: bleeding requiring blood transfusion was reported in three patients, deep vein thrombosis was diagnosed in one patient after removal of the ECCO_2_R catheter, one patient experienced pneumothorax due to catheter insertion, and one died from hemorrhage when the iliac vein was perforated during ECCO_2_R catheter placement.

Del Sorbo et al. [4] compared 25 patients with aeCOPD treated with NIV + VV ECCO_2_R versus 21 historical controls treated with NIV alone with regard to the cumulative incidence of intubation. They reported that ECCO_2_R with a 14-Fr dual-lumen catheter and blood flow rates of 177–333 mL/min not only improved respiratory acidosis but also reduced the need for intubation by 75% (12% vs. 33%; *p* = 0.047) and significantly reduced the in-hospital mortality (8% vs. 35%; *p* = 0.035). However, this came with a cost of 52% prevalence of ECCO_2_R-related side effects and led the authors to suggest the end point of future studies should be long-term mortality.

Braune et al. [43] in the ECLAIR study showed that IMV was avoided in 56% of cases treated with VV ECCO_2_R, which was associated with a high incidence of complications. However, in this study, there was an inclusion of patients with relative contraindications to NIV, and there was an unexpectedly high incidence of hypoxemic patients [44].

Finally, Morelli et al. [45] confirmed the efficacy of VV ECCO_2_R (with a flow rate of 250–450 mL/min through a 13-Fr dual-lumen cannula) in reducing the PaCO_2_ in a series of 30 patients with acute hypercapnic respiratory failure due to aeCOPD, who refused endotracheal intubation after failing NIV. The duration of ECCO_2_R was 2–16 days, and it was possible to prevent endotracheal intubation in 27 patients.

### ECCO_2_R to facilitate weaning from IMV

Cardenas et al. [46] made the first attempt to use modern ECLS components for VV ECCO_2_R in a patient with aeCOPD. They demonstrated a successful reduction in PaCO_2_, minute ventilation, and ventilator pressures.

Burki et al. [42] in a subgroup of 11 patients receiving IMV, ECCO_2_R allowed the weaning from mechanical ventilator in only three patients.

Abrams et al. [3] reported five older patients (age 73 ± 8.7 years) with aeCOPD who failed NIV, requiring IMV. After an average of 16.5 ± 5.9 h of IMV, ECCO_2_R was initiated. By using a dual-lumen cannula (20–23 Fr) with blood flow rates of 1–1.7 Lt/min, with a sweep gas flow from 1 to 7 L/min, they were able to extubate all five patients within 24 h of treatment (median duration of MV post ECCO_2_R = 4 h, range 1.5–21.5 h). Once extubated, patients were rehabilitated while on ECCO_2_R, with a mean time to ambulation of 29.4 ± 12.6 h after ECCO_2_R. Moreover, all patients survived to hospital discharge.

Roncon-Albuquerque Jr. et al. [47] using a pediatric VV ECMO system (with blood flow rates of 0.9 L/min through a 19 Fr dual-lumen cannula placed in the right jugular vein) in two patients with aeCOPD reported early extubation after 72 h and patient mobilization out of bed at day 6.

#### Future studies on ECCO_2_R for COPD

More data will be forthcoming on the application of ECCO_2_R in the management of patients with COPD exacerbations from a number of ongoing or planned clinical trials (Table 2).

**Table 2:** Ongoing or completed clinical studies of ECCO_2_R in COPD

ClinicalTrials.gov number	Title	Type of study	Hypothesis/primary outcome	Estimated enrollment	Device	Status
ECCO_2_R to avoid mechanical ventilation						
NCT02564406	Extracorporeal CO_2_ removal in hypercapnic patients	Interventional single-group trial	Retrospectively assess the efficacy and safety of noninvasive ventilation-plus-extracorporeal CO_2_ removal in patients who fail NIV and refuse endotracheal intubation Primary outcome: Number of patients who avoided endotracheal intubation	35 patients	ProLUNG [Estor]	Completed
NCT03692117		Prospective cohort study	Primary outcome: Incidence of avoiding endotracheal intubation	30 patients	Not specified	Recruiting
ECCO_2_R as an alternative or adjunct to invasive mechanical ventilation						
NCT03255057	Extracorporeal CO_2_ removal for mechanical ventilation avoidance during acute exacerbation of COPD (VENT-AVOID)	Multicenter randomized controlled trial	ECCO_2_R can be safely used to avoid or reduce time on invasive mechanical ventilation compared to COPD patients treated with standard-of-care mechanical ventilation alone Primary outcome: Ventilator-free days at day 60 from randomization	500 patients	Hemolung	Recruiting
ECCO_2_R physiological studies						
NCT02586948	Physiological study of minimally invasive ECCO_2_R in exacerbations of COPD requiring invasive mechanical ventilation (EPHEBE)	Interventional single-group trial	The addition of minimally invasive ECCO_2_R is likely to limit dynamic hyperinflation in COPD patients requiring invasive mechanical ventilation for an acute exacerbation while improving gas exchange Primary outcome: PEEPi at baseline and after ECCO_2_R by the device and adjustment of ventilator settings, expressed in cmH20	12 patients	Hemolung	Completed
NCT02590575		Interventional single-group trial	Test the effectiveness of a membrane gas exchange device in the veno-venous circulation of continuous renal replacement therapy for the purpose of CO2 elimination and pH compensation The primary outcome is the modification of the PaCO_2_ and/or the ventilator settings (tidal volume VT and plateau pressure Pplat)	20 patients	Prismalung	Completed

Despite the strong physiological rationale, the existing data are not sufficient to support the routine use of ECCO_2_R in patients with aeCOPD, as randomized controlled trials investigating the efficacy of ECCO_2_R in improving important patient centered outcome are lacking, and the intervention is associated with a high rate of complications.

Furthermore, the relevant incidence of ECCO_2_R-related complications considerably affects the choice of the target patient population of randomized controlled trials, and hence their inclusions and exclusion criteria. The application of ECCO_2_R to prevent IMV in aeCOPD patients at high risk of NIV failure has a remarkable potential clinical impact, but exposes a number of patients, who will not require IMV, to the unnecessary risk of ECCO_2_R-related complications. The application of ECCO_2_R in aeCOPD patients intubated after NIV failure to accelerate liberation from IMV exposed patients simultaneously to the complications of two invasive treatments. In both scenarios, given the high mortality rate associated with IMV in this obviously vulnerable patient population, these studies should be powered to demonstrate a mortality benefit.

The development of new ECCO_2_R technology with less associated complications will allow the study of ECCO_2_R also in patients with milder severity of aeCOPD or even in stable COPD patients to prevent the occurrence of exacerbations.

### Severe acute asthma

Asthma is an inflammatory disorder of the airways characterized by airway hyperactivity with bronchospasm, mucosal swelling, and mucus production. The standard treatment of severe acute asthma consists of measures to reverse airflow obstruction. β2 agonists and steroids are the mainstays of treatment [12]. Other available adjunct therapies including anticholinergics, magnesium sulfate, methylxanthines, ketamine, and heliox have been utilized with varying results [48].

Despite advances in asthma therapy, asthma mortality has remained stable in recent years. One reason is the occurrence of status asthmaticus, which can be unresponsive to initial treatment and may lead to hypercapnic respiratory failure despite maximal therapy, and in the most severe cases requires IMV.

Approximately, 4% of all patients hospitalized for acute asthma require IMV, which is associated with increased in-hospital mortality compared with patients who do not require mechanical ventilation (7 vs. 0.2%) [49].

Although necessary, mechanical ventilation may aggravate alveolar hyperinflation as it was described above. To prevent these potential detrimental effects, ECCO_2_R has been applied as rescue therapy.

ECCO_2_R as an adjunct to IMV for refractory asthma was first reported in 1981 [50]. Subsequently, several case series have been reported (Table 3) [51–55]. In the international Extracorporeal Life Support Organization (ELSO) registry, the use of ECMO for asthma has been reported in 24 adult patients between 1986 and 2006. Hypercapnia, rather than hypoxemia, was the main gas exchange derangement treated with ECMO, suggesting that a less invasive approach, such as low flow ECCO_2_R, could also be suitable in these cases. Indeed, the use of ECCO_2_R in patients with asthmatic exacerbation has been reported, although in a limited number of cases.

**Table 3:** Case series of ECCO_2_R for near fatal asthma

References	ECCO_2_R technique	Major findings
Sakai et al. [52]	Extracorporeal lung assist (ECLA); 22 Fr drainage and 18 Fr return femoro-femoral cannula with a median blood flow rate of 1.7–2 L/min	23 year old Gas exchange with IMV before ECCO_2_R: pH 7.02, paCO_2_ 100 mmHg, PaO_2_ 50 mmHg (FiO_2_ 100%) Weaning achieved after 20 h of ECLA was commenced Extubation 2 days after ECLA No complications reported
Elliot et al. [53]	Femoral AV pumpless extracorporeal lung assist (PECLA) 15-Fr arterial cannula and 17-Fr venous cannula with a mean extracorporeal blood flow of 1.5 L/min	Case 1: 74 year old. Gas exchange with IMV before ECCO_2_R: pH 6.87, paCO_2_ 147 mmHg. Extubation after 48 h of ECLA. Complications: Coagulation of membrane that needed changing. Bleeding through femoral artery Case 2: 52 year old. Gas exchange with IMV before ECCO_2_R: pH 7.2, paCO_2_ 130 mmHg. ECCO_2_R duration: 5 days Extubated on intensive care day 11. No complications reported
Jung et al. [54]	Femoral AV pumpless extracorporeal lung assist (PECLA) 15-Fr arterial cannula and 17-Fr venous cannula with a mean extracorporeal blood flow of > 1.5 L/min	42 year old No gas exchange before IMV reported. Patient successfully extubated and transferred from the ICU on day 14 of admission No complications reported
Brenner et al. [51]	Dual-lumen catheter 20–23 Fr bicaval, inserted into the right internal jugular vein with blood flow of 1.3 to 1.8 L/min	Case 1: 48 years old. Gas exchange with IMV before ECCO_2_R: pH 6.94, paCO_2_ 147 mmHg, PaO_2_ 416 mmHg (FiO_2_ 100%). Successfully extubated while on ECCO_2_R and discharged from ICU. No complications reported Case 2: 59 years old. Gas exchange with IMV before ECCO_2_R: pH 7.12, paCO_2_ 78 mmHg, PaO_2_ 112 mmHg (FiO_2_ 100%). ECCO_2_R duration: 9 days. Ventilator support discontinued on day 28 due to critical illness neuromyopathy
Schneider et al. [55]	Awake dual-lumen catheter 22 Fr bicaval, inserted into the right internal jugular vein with blood flow of 0.6–1.5 L/min	67 years old Gas exchange before ECCO_2_R (on NIV): pH 7.24, paCO_2_ 61 mmHg, PaO_2_ 289 mmHg (FiO_2_ 100%) Thirty-four hours after initiating ECCO_2_R, the patient was weaned entirely from NIV, and the cannula could be removed without any complication. On day 4, the patient was discharged from the ICU without the need for supplemental oxygen and 6 days later, discharged from hospital without any impairment

*IMV* invasive mechanical ventilation, *NIV* noninvasive mechanical ventilation

## Complications

Although ECCO_2_R seems to be effective in improving or mitigating hypercapnic acidosis and possibly in reducing the rate of endotracheal intubation, its use is associated with a range of vascular, hematological, and other complications (Table 4).

**Table 4:** ECCO_2_R-related complications

Patient-related complications	Anticoagulation-related bleeding Hemolysis Heparin-induced thrombocytopenia Acquired coagulopathy Recirculation
Catheter-related complications	Catheter-site bleeding Catheter malposition, dislodgement or kinking Catheter infection Vascular occlusion Thrombosis Hematoma, aneurism, pseudoaneurysm formation
Device-related complications	Pump failure Oxygenator failure Heat-exchanger malfunction Clot formation Air embolism

Arterial cannulation is associated with higher risk than venous catheterization, with specific complications including distal limb ischemia, compartment syndrome of the lower limb requiring fasciotomy or limb amputation, as devastating consequences [18].

The occurrence of bleeding events is the most frequent complication of ECCO_2_R. The low flow renders systemic anticoagulation mandatory, increasing the risk of significant bleeding including cerebral, gastrointestinal, and nasopharyngeal bleeds. In the studies on ECCO_2_R for COPD to date, the rate of clinically significant hemorrhagic complications ranges between 2% and 50% [56].

Thrombocytopenia is also commonly observed, as well as hemolysis.

Conversely, thrombus formation is higher at lower blood flow rates because of increased exposure time to the membrane lung and circuit. Clots may detach and enter the patient’s bloodstream, plugging the membrane or obstructing the cannula if anticoagulation is not achieved.

## Conclusion

In the past, ECCO_2_R was a complex technique requiring intensive monitoring and surgical expertise. Due to a high rate of complications, it was avoided by all but few high expertise centers. With newer simplified systems, ECCO_2_R devices can be easily used and can be initiated by most intensivists. However, given the lack of conclusive clinical evidence and the relatively high rate of associated complications, its use should be restricted to investigational applications in specific cohorts of patients.

In summary, minimally invasive ECCO_2_R appears very promising for patients with acute exacerbation of obstructive diseases refractory to conventional treatment, but systematic evaluation is needed to prove its clinical efficacy.

## Data Availability

Data sharing not applicable to this article as no datasets were generated or analyzed during the current study.

## Abbreviations

AV-ECCO_2_R: arterio-venous extracorporeal carbon dioxide removal
COPD: chronic obstructive pulmonary disease
CO_2_: carbon dioxide
DHI: dynamic hyperinflation
ECCO_2_R: extracorporeal carbon dioxide removal
ECMO: extracorporeal membrane oxygenation
IMV: invasive mechanical ventilation
NIV: noninvasive ventilation
PECLA: pumpless extracorporeal lung assist
PEEP: positive end-expiratory pressure
PLP: polypropylene
PMP: poly-4-methyl-1-pentene
VV-ECCO_2_R: veno-venous extracorporeal carbon dioxide removal
